# Hypoxic culture of umbilical cord mesenchymal stem cell-derived sEVs prompts peripheral nerve injury repair

**DOI:** 10.3389/fncel.2022.897224

**Published:** 2023-03-09

**Authors:** Ziying Zhu, Yujun Zhang, Zhihua Huang, Haojie Hao, Muyang Yan

**Affiliations:** ^1^The First Medical Center of Chinese PLA General Hospital, Beijing, China; ^2^Beijing Key Laboratory of Protein Posttranslational Modifications and Cell Function, Department of Biochemistry and Biophysics, Peking University Health Science Center, Beijing, China

**Keywords:** umbilical cord mesenchymal stem cells, peripheral nerve injury (PNI), sEVs, regeneration, Schwann cells, repair, hypoxia

## Abstract

**Introduction:**

Repair and regeneration of the peripheral nerve are important for the treatment of peripheral nerve injury (PNI) caused by mechanical tears, external compression injuries and traction injuries. Pharmacological treatment can promote the proliferation of fibroblasts and Schwann cells (SCs), which longitudinally fill the endoneurial canal and form Bungner’s band, helping the repair of peripheral nerves. Therefore, the development of new drugs for the treatment of PNI has become a top priority in recent years.

**Methods:**

Here, we report that small extracellular vesicles (sEVs) produced from umbilical cord mesenchymal stem cells (MSC-sEVs) cultured under hypoxia promote repair and regeneration of the peripheral nerve in PNI and may be a new therapeutic drug candidate.

**Results:**

The results showed that the amount of secreted sEVs was significantly increased in UC-MSCs compared with control cells after 48 h of culture at 3% oxygen partial pressure in a serum-free culture system. The identified MSC-sEVs could be taken up by SCs in vitro, promoting the growth and migration of SCs. In a spared nerve injury (SNI) mouse model, MSC-sEVs accelerated the recruitment of SCs at the site of PNI and promoted peripheral nerve repair and regeneration. Notably, repair and regeneration in the SNI mouse model were enhanced by treatment with hypoxic cultured UC-MSC-derived sEVs.

**Discussion:**

Therefore, we conclude that hypoxic cultured UC-MSC-derived sEVs may be a promising candidate drug for repair and regeneration in PNI.

## 1. Introduction

Peripheral nerve injury (PNI) is a common surgical disease and a difficult condition for tissue trauma repair. There are many clinical factors related to the development of PNI, such as tumor resection, trauma, and systemic diseases. PNIs account for 2.8% of cases of neurological disorders, and the prevalence is increasing every year (Scholz et al., 2011; Deuis et al., 2017). After the injury, peripheral nerves need to be repaired and rebuilt within a certain period of time; otherwise, the function of the organ or tissue they control will be lost, resulting in a high disability rate and a great social burden. In addition to timely nerve sutures, autologous nerve transplantation is generally used to clinically treat PNI, but the neural function is lost, a surgical scar remains, and a donor must be found. Upon additional incision, due to graft dysfunction of the innervation area and a series of complications, a repair cannot be guaranteed. Therefore, it is of great importance to explore more ideal treatment methods to promote the repair and regeneration of damaged peripheral nerves after trauma.

During regeneration after the degeneration of the peripheral nerve, a large number of macrophages gather to engulf denatured axons, disintegrate myelin sheath fragments and dead Schwann cells (SCs), and form empty endoneurium tubes so that regenerated axial buds can reach target organs along the endoneurial tubes (Lee and Wolfe, 2000). Macrophages in this region release growth factors that stimulate the proliferation of SCs and fibroblasts. SCs longitudinally fill the endoneurial canal to form Bungner’s bands (Pfister et al., 2011).

Schwann cells exhibit strong plasticity during the repair of PNI. After PNI, SCs demyelinate and differentiate into repair SCs. This phenotypic SC-transformation process is called dedifferentiation. Repair SCs initiate the nerve regeneration process, downregulate myelination genes, and activate genes that negatively regulate myelination (Skotland et al., 2017), thereby removing damaged axons and myelin sheath fragments and creating a favorable environment for nerve regeneration. c-JUN, mitogen-activated protein kinase (MAPK), and other signaling pathways and transcriptional regulatory factors are involved in this process (Familtseva et al., 2019). Therefore, the early selection of drugs to regulate the status of SCs in PNI is crucial for timely and effective recovery from PNI.

Small extracellular vesicles (sEVs) derived from stem cells carry mRNA, ribosomal RNA, long non-coding RNA, and some cytokines. sEVs participate in exchanging various information and substances between cells through horizontal transfer and carry out biosynthesis and absorption, immune monitoring, microenvironmental modification, inflammatory regulation, etc.; thus, sEVs promote tissue repair and regeneration (Colombo et al., 2014). The repair abilities of sEVs from different stem cell types differ. Compared with other types of stem cells, human umbilical cordderived mesenchymal stem cells (MSCs) have the strongest neural differentiation capacity, so sEVs derived from human umbilical cord-derived MSCs may promote neural tissue repair better than sEVs derived from other stem cell types (Hendrijantini and Hartono, 2019). Stem cells secrete fat-soluble sEVs, which more freely through the vessel wall and the blood–brain barrier, have good stability, are conducive to storage and transport, can be used as good immunogenic drug carriers, and increase the biological effects of loaded drugs; furthermore, stem cell-derived sEVs secreted by the body promote extracellular matrix formation. These qualities make stem cell-derived sEVs a new “cell-free” strategy for nerve tissue repair. Many studies have observed that stem cell-derived sEVs can accelerate peripheral nerve regeneration after injury, but the molecular mechanism of nerve regeneration has not been clarified, and basic information on stem cell-derived sEVs remains to be further studied (Ludwig et al., 2019).

A change in oxygen volume fraction will lead to a corresponding change in cell physiological function. The average oxygen volume fraction in normal organisms is 5%, but most studies on stem cells have been carried out under normoxic (volume fraction 21% O_2_) conditions (Nocera and Jacob, 2020). Studies have shown that adipose MSCs can survive in a hypoxic environment under physiological conditions, so the cultivation of adipose MSCs under a hypoxic environment *in vitro* is more consistent with the *in vivo* environment (Costales et al., 2019). Here, we investigated whether sEVs derived from umbilical cord MSCs (MSC-sEVs) can promote the repair and regeneration of PNI as a new candidate drug treatment.

## 2. Materials and methods

### 2.1. Cell culture

Human umbilical cord-derived mesenchymal stem cells were obtained from the PLA General Hospital. Cells were grown in a serum-free culture system in Nuwacell™ ncMission hMSC medium (RP02010, Nuwacell, China).

Human umbilical cord-derived mesenchymal stem cells were inoculated in T175 flasks (708003, Nest) at a density of 0.6 × 10^3^ cells/cm^2^ and incubated in a 5% CO_2_ atmosphere at 37°C for 48 h.

Schwann cells were routinely cultured using DMEM (MB3373, Gibco, USA) with 10% fetal bovine serum (FBS, 10099141C, Gibco, USA) and 1% penicillin–streptomycin (Bio, RoYee). in 5% CO_2_ at 37°C.

Hypoxic training: Hypoxic incubated UC-MSCs were inoculated in an incubator with a normal oxygen content for 24 h and then transferred to a hypoxic cellular incubator with 3% O_2_ (Baker Ruskinn, PhO2x Box) for another 48 h.

The final concentration of hypoxic sEV cell culture administration was 0.1 g/L.

### 2.2. Extraction and identification of sEVs

Pretreatment: The cell supernatant was collected with dead cells, and debris was removed using a 0.45-μm cell filter (343103, Nest), after which the supernatant was centrifuged at 10,000 × *g*. The supernatant was preconcentrated using a 3,000 MWCO PES membrane (VF20P9, Sartorius, German), and sEVs were extracted using a total exosome isolation reagent (4478359, Invitrogen).

Extraction: Reagent was added to the concentrated cell supernatant and incubated overnight at 4°C. The precipitated sEVs were recovered by centrifugation at 4°C and 10,000 × *g* for 60 min.

The sEV morphology was photographed using transmission electron microscopy (TEM). sEVs were diluted to the appropriate concentration using PBS, stained with 2% uranyl acetate for 1 min, and then transferred to a carbon-coated copper grid. sEVs in a single field of view were photographed by TEM (Hitachi, Japan, H-7500) to assess their morphology. The molecular biomarker proteins (CD9, CD81, and TSG101) were chosen for analysis with Western Blotting. sEVs (2,000×) were diluted with DPBS and then placed in a syringe tube. Standard operating procedures with default parameters were used. The size and particle number of the sEVs were analyzed with a Flow NanoAnalyzer (NanoFCM, China, U30).

### 2.3. Small extracellular vesicle labeling and uptake

After SCs were labeled with a fluorophore, UC-MSCs sEVs were labeled using the PKH67 Green Fluorescent Labeling Kit (BB-441,112, Bestbio). Then, they were incubated with SCs at 37°C for 12 h. After that, the cells were examined under a Zeiss 880 confocal laser scanning microscope at ×200 magnification.

### 2.4. Irradiation

To research the effect of sEVs in restoring cell growth and proliferation, we used 10 Gy as the irradiation dose to irradiate the cells on the first day after cell apposition and changed the fluid after 24 h. sEVs in the experimental group were added at the time of fluid change. The rest of the culture conditions followed the normal culture practice.

### 2.5. Colony formation assay

Cells in both control and experimental groups were seeded in six-well plates at 500 cells/well in triplicate and maintained under standard conditions for 10–14 days. After fixation, the cells were stained with a crystal violet staining solution (Sigma-Aldrich, China). Colonies were counted and imaged under a microscope.

### 2.6. Cell proliferation assay

Cell viability was detected using a Cell Counting Kit-8 (CK04, Dojindo Laboratories, Japan) assay. Cells were seeded at 3,000 cells/well in 96-well plates in triplicate. Then, 100 μL of DMEM containing 10 μL of WST-8 reagent was added to each well every 24 h. Then, the plates were incubated at 37°C for 1 h, and the optical absorbance of each well at 450 nm was measured using a microplate reader (Multiskan FC, Thermo, USA). A total of 10^3^ cells in the wild-type or experimental group were inoculated in six-well plates.

### 2.7. Cell scratch test

When cells seeded in a six-well plate reached confluence, a single scratch was made using a sterile 10-μl pipette tip. The cells were then incubated with an FBS-free culture medium. Images of the scratches were captured at 0, 24, and 48 h with an inverted microscope (S9E, Leica, German) at 100× magnification. The width of the scratch was analyzed using ImageJ software.

### 2.8. Western blotting

Western blotting was performed in a standard manner as described in a prior study (Duarte-Moreira et al., 2018). The following primary antibodies diluted 1:1,000 were used: Anti-c-JUN (ab40766, Abcam, UK), anti-SOX2 (ab92494, Abcam, UK), anti-ERK1 + ERK2 (ab184699, Abcam, UK), and anti-ZEB2 (ab191364, Abcam, UK); anti-β-actin (Sigma, AC-15) diluted at 1:5,000 was also used. Rabbit IgG HRP-linked secondary antibody (NA934-1 ml, GE) and mouse IgG HRP-linked whole antibody (NA931-1 ml, GE) antibody (NA931-1 ml, GE) were used at a dilution of 1:10,000.

### 2.9. ELISA

ELISA test was performed according to the instruction method. The following ELISA kits were: mouse glial cell line-derived neurotrophic factor (GDNF), ELISA kit (CSB-E07341m, CUSABIO), mouse neurotrophin 3 (NT-3), ELISA kit (CSB-E04687m, CUSABIO), and mouse nerve growth factor (NGF), ELISA kit (CSB-E04684m, CUSABIO).

### 2.10. RNA isolation, cDNA synthesis, and qPCR

Total RNA was isolated from SCs using a ReverTra Ace^®^ qPCR RT Kit (FSQ-101, Toyobo, Osaka, Japan). The cells were placed directly into TRIzol reagent (15596-026, Invitrogen, Carlsbad, CA, USA) and then precipitated by chloroform and alcohol purification. In a 20-μL reaction volume, 2× SYBR Master Mix was combined with forward and reverse primers at 1 μM each and amplified on an ABI 7,500 system (Applied Biosystems, Foster City, CA, USA) over 40 cycles at 95°C for 10 s and 60°C for 30 s. After amplification, melting curve analysis was performed according to the manufacturer’s protocol, and the software provided with the instrument was used to determine relative expression, which was normalized to the expression of glycerol triphosphate dehydrogenase (GAPDH). Relative gene expression was analyzed in triplicate.

1. Animal experiment

The Institutional Animal Care and Use Committee of Peking University approved all animal studies. A spared nerve injury (SNI) model was established in male C57BL/6J mice (18–20 g). The mice were adequately acclimated to the experimental environment prior to surgery.

2. Spared nerve injury model: Under anesthesia with 10% chloral hydrate (0.1 ml/100 g i.p.), the skin of the right posterior thigh was incised, and the sciatic nerve and its three branches behind the femur were exposed through the biceps femoris muscle. The nerve was fully compressed for 60 s using X-gauge surgical forceps, and the nerve was completely truncated while ensuring the integrity of the epineural membrane. Hypoxic sEVs were administered at a dose of 2.76 mg/g body weight. The sEVs are administered by local subcutaneous injection.

### 2.11. Hematoxylin and eosin (HE) staining

Mice were killed by cervical dislocation at 0, 24, 48, and 72 h after nerve injury. The sciatic nerve was isolated, fixed in 4% paraformaldehyde for 24 h, and dehydrated in a graded alcohol series. After treatment with xylene, the specimens were embedded in paraffin blocks. Longitudinal serial sections of 4-μm thickness and 10-mm length were obtained from the sciatic nerve. The sections were then subjected to HE staining according to standard procedures.

### 2.12. Methylamine blue staining

The sciatic nerve sections fixed in 4% paraformaldehyde were removed and rinsed three times with DPBS. A prewarmed 1% toluidine blue solution (G1220, Solarbio, China) was added, and the sections were stained in a 50°C water bath for 20–30 min. Graded alcohol solutions were used sequentially to decolorize the sections, and the decolorization effect was continuously monitored. The nuclei were light blue, and the background was basically colorless.

### 2.13. Von frey test

Mechanical allodynia in mice was assessed by the previously described von Frey test (Nishio et al., 2002). Two days and 1 day before surgery, the mice were placed in a red plastic cylinder and placed on a wire mesh table. Before the test, the mice get used in the cylinder for 15 min. The test was applied to the middle of the plantar surface of the ipsilateral and contralateral paws of mice. Licking or shaking the paw was considered a positive response, while the absence of the aforementioned responses within 5 s was considered a negative response. The mechanical threshold was defined as “the minimum force that induced at least 3 paw retraction reflexes in 5 consecutive trials” and is expressed as paw withdrawal threshold (PWT) in grams.

### 2.14. Statistics

Data collected from at least three different experiments for repeats were statistically analyzed and are expressed as the mean ± standard deviation (SD). Data analysis was performed using Prism 8.0 software (GraphPad, San Diego, CA, USA). Comparisons between the two groups were performed by *t*-test. Comparisons between unpaired data from two groups were performed by Student’s *t*-test. A *P*-value of less than 0.05 indicated statistical significance.

## 3. Results

### 3.1. Hypoxic culture promoted UC-MSC secretion of sEVs with an intact structure

**FIGURE 1:**
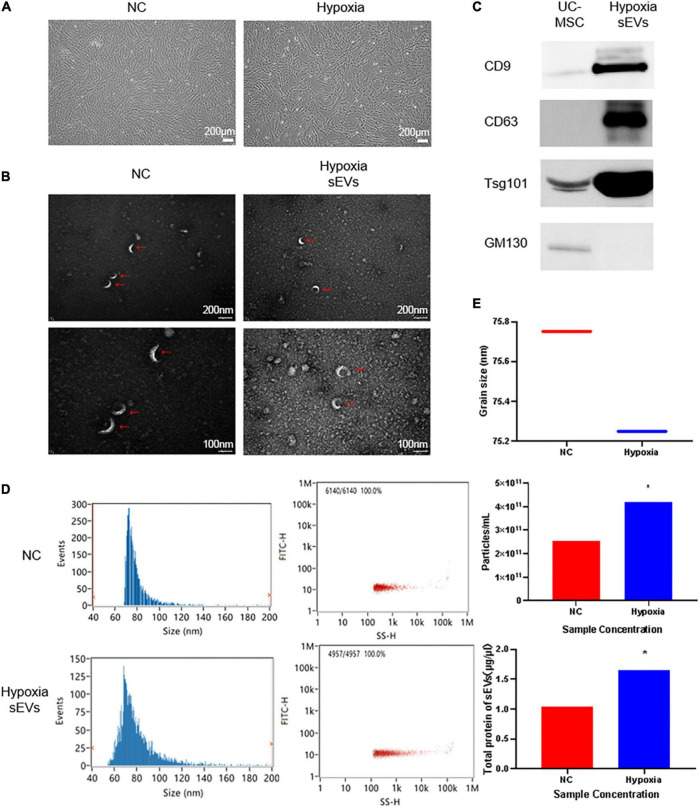
Identification of UC-MSC-derived sEVs in hypoxic culture: **(A)** Cell culture: UC-MSCs under normoxic culture and hypoxic culture underwent microscopic examination to assess their morphology. UC-MSCs were seeded in T175 flasks at a density of 0.6 × 10^6^ cells/cm^2^, cultured under different conditions for 72 h, and then observed under a microscope. The cell viability was greater than or equal to 98%, and the cell convergence rate was greater than 85%. **(B)** Transmission electron microscopy (TEM) of sEVs to assess morphology: upper, 20,000× field of view; lower, 2× zoomed image. **(C)** Determination of a characteristic antigen on sEVs: Intact UC-MSCs were used as a control, and the extracted hypoxic cultured UC-MSC-derived sEVs exhibited characteristic surface antigen expression. GM130 was used as a negative control marker. Statistical significance, *p* < 0.05. **(D)** Nano-FCM was used to determine the sEV particle distribution and scattered light parameters; the main peak distribution between 30 and 150 nm and median of 75 ± 0.25 conformed to the characteristic diameter distribution of sEVs. **(E)** Determination of the sEV concentration and particle number. *Significant difference.

Human umbilical cord-derived mesenchymal stem cells and their cell products are of great interest because of their stemness and therapeutic potential. We inoculated T175 flasks with cells at a density of 0.6 × 10^5^ cells/cm^2^ and transferred the cells to a 3% hypoxic incubator after 24 h of normoxic culture for continued culture for 48 h. The morphology of UC-MSCs cultured in hypoxia is shown (Figure 1A). The fusion rate of UC-MSCs cultured in hypoxia was greater than or equal to 80%, and the UC-MSCs exhibited an intact morphology and a strong refractive index. The expansion curve of UC-MSCs cultured in hypoxia was lower than that of cells in the NC (abbr: Normoxia Condition) group cultured in normoxia, but the difference was not statistically significant. After collecting the cell culture supernatant to initially remove the debris, such as dead cells, sEVs were extracted. Using TEM with a 20,000× magnification (Figure 1B), both the hypoxic culture group and NC group contained sEVs with a uniform diameter distribution. A complete cup-shaped morphology was observed under 40,000× magnification. Western blot detection of characteristic antigens on the surface of the sEVs confirmed that sEVs in hypoxic culture expressed CD9, CD63, and Tsg101 (Figure 1C). The particle size distribution and the content of sEVs in both groups were measured using nano-flow cytometry (Nano-FCM) (Figure 1D). The BCA assay showed that the protein content of the sEVs extracted from the supernatant of hypoxic cultured cells was 30% higher than that of sEVs extracted from the supernatant of the control group. The number of particles was approximately two times as high as that of the normoxic culture group, while the particle distribution diameter and number of particles detected in the channel (Figure 1E) were consistent with the consensus range of 30–150 nm for sEVs. Thus, we confirmed that the hypoxic culture of UC-MSC stimulated the cells to produce more sEVs and that this conditioned stimulation did not affect the integrity of the sEV structure.

### 3.2. Small extracellular vesicles derived from hypoxic cultured UC-MSCs could be taken up by neuronal cells and regulate the expression level of neural repair-like factors

**FIGURE 2:**
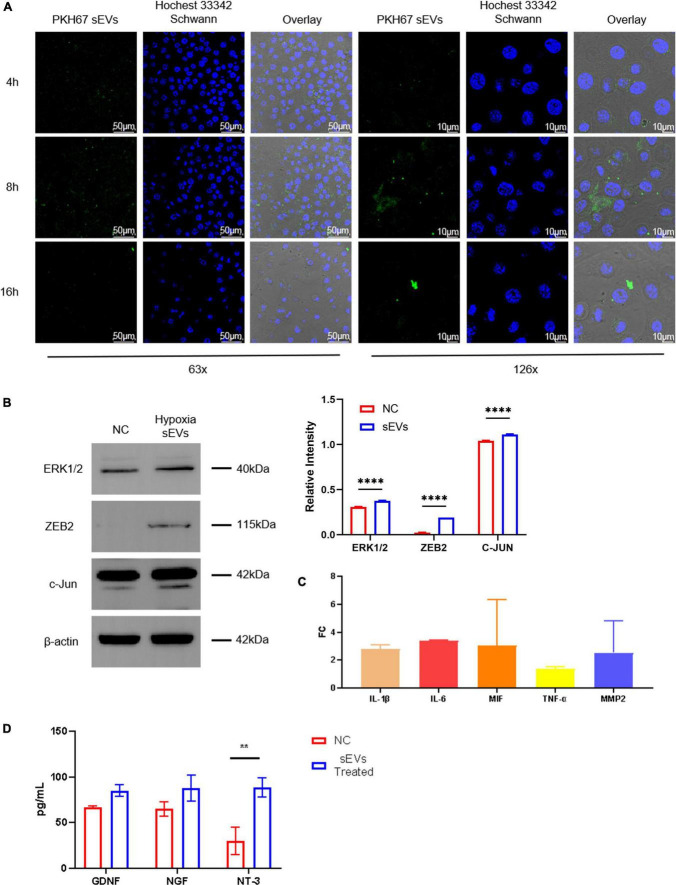
Cellular localization and uptake of fluorescently labeled sEVs with SCs. **(A)** Cellular colocalization: SP8 was used to photograph sEVs prelabeled with PKH67 (green fluorescence) with Hoechst 33,342 (blue fluorescence)-stained SC nuclei. **(B)** Western blot: Detection of ERK1/2, ZEB2, and c-JUN expression levels in NC Schwann cells and after 48-h treatment with hypoxia sEVs. **(C)** Real-time PCR: Inflammatory and other restoration-related factors (IL-1β, IL-6, TNF-α, etc.) were detected. **(D)** ELISA test: GDNF NDF and NT-3 in the supernatant of Schwann cell preparation. Statistical significance, *p < 0.05,**p < 0.01, and ****p < 0.0001. *Significant difference.

To determine whether sEVs could be taken up and utilized by neuronal cells, we co-cultured UC-MSC-derived sEVs with SCs as the target. SCs were spread in a confocal dish and monolayers of an appropriate density formed after 24 h of culture. At this time, PKH67-labeled sEVs, which had previously been extracted, stained, and purified again, were added to the confocal dish at a concentration of 0.1 mg/ml, and the SCs were then cultured away from light. sEVs were co-cultured with SCs using green fluorescent labeled sEVs, and images were taken at three consecutive time points starting at 4 h. The uptake of sEVs by SCs was observed (Figure 2A). The fluorescence intensity of sEVs in the field of view peaked at 8 h and decreased again at 16 h to a level lower than that at 4 h. Observation at 2× magnification showed scattered green fluorescence in the field of view at 4 h, which suggested that the sEVs were intact and distributed around the cells, with a small amount of fusion; the increase in overall fluorescence intensity in the field of view at 8 h was observed because the sEVs were intact. In addition, the initial disintegration of green fluorescence in the cytoplasm suggested that some of the ingested sEVs had fused into the membrane of the SCs, but intact sEVs were still continuously recruited. At 16 h, the overall fluorescence intensity had decreased, but intact sEVs appeared scattered in individual cells, indicating that the sEV content in the co-culture system had significantly decreased after the peak of uptake, but the cells could still recruit sEVs from the culture system. The cells were able to recruit and take up sEVs derived from UC-MSCs in hypoxic culture, and peak uptake was observed at 8 h. To further explore the effect of hypoxic sEVs on SCs, experimental cells were added with a concentration of 0.1 μg/μL of hypoxic sEVs and incubated for 48 h before the Western Blot assay. The results showed that the expression levels of ZEB2 ERK1/2 c-JUN, a key factor that plays an activating role in neural repair pathways, were all upregulated to different degrees. This indicates that hypoxic sEVs have the effect of inducing repair in SCs. It indicates that hypoxic sEVs have the effect of prompting the activation of SC repair function (Figure 2B). An important component of the injury response is the activation of the immune response, which consists of the upregulation of cytokines, including IL1-β (Costales et al., 2019). Elevated expression of IL-6 and TNF-α has also been demonstrated to occur early in sciatic nerve injury in rats (Duarte-Moreira et al., 2018). These inflammatory factors are not only indicative of inflammation but also are involved in the activation of anti-inflammatory pathways (Nishio et al., 2002; Muscella et al., 2020). Macrophage movement inhibitory factor (MIF) is a pluripotent cytokine involved in inflammatory and immune responses and cell growth (Rylova et al., 2012). TGF-β1 plays an important regulatory role in the migration of residual SCs after injury and subsequent repair and regeneration. This repair-promoting function has been shown to be mediated by MMP-2 (Chung et al., 2009). Accordingly, we co-cultured sEVs derived from hypoxic cultured UC-MSCs and SCs and collected the SCs at 48 h for real-time fluorescence quantification (Figure 2C). However, the mRNA levels of all these factors were not significantly different from the control group. The level of NT-3 secretion in neurotrophic secretory factor was significantly increased after 48 h of treatment with hypoxic sEVs (Figure 2D). Accordingly, we can know that Schwann can activate repair-related signaling pathways and increase NT-3 secretion levels under the short-time effect of hypoxic sEVs.

### 3.3. Small extracellular vesicles derived from hypoxic cultured UC-MSCs rescued neuronal cell injury and maintained cell viability

**FIGURE 3:**
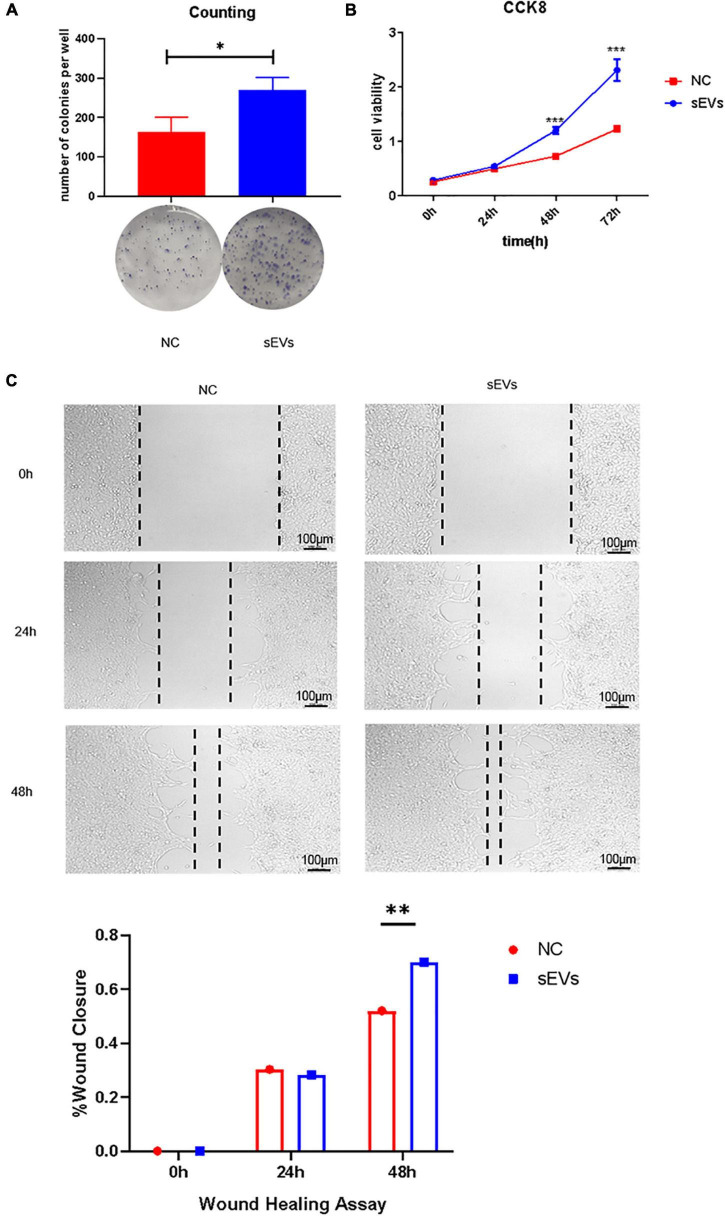
Protective effect of sEVs derived from hypoxic cultured UC-MSCs against cell damage. **(A)** Colony formation assay: 700 cells/well were preliminarily grown in a monolayer and irradiated after reaching confluence. The medium was changed daily, and the medium in the experimental group was supplemented with 0.1 mg/ml of hypoxic cultured sEVs. **(B)** Cell viability assay (CCK-8). **(C)** Scratch assay. The growth of the experimental and control groups was observed over a 72-h cycle. Statistical significance, *p* < 0.05. *, ^**^, ^***^Significant difference.

We used irradiated (10 Gy) SCs to construct a cell injury model and observed the effect of sEVs derived from hypoxic cultured UC-MSCs in restoring cell growth and proliferation. A clonogenic assay (Figure 3A) confirmed that SCs with a reduced expansion rate after injury were partially protected from division after continuous cell culture with sEVs derived from hypoxic UC-MSC cultures (0.1 μg/μL). There was a significant difference in the number of colonies formed compared to that in the control group, which received only irradiation.

Cell viability assays demonstrated a significant increase in cell viability in the sEV group (Figure 3B). The addition of sEVs from hypoxic cultured UC-MSCs promoted scratch healing under equivalent culture conditions as confirmed by scratch experiments (Figure 3C). It revealed that the sEVs protected cell growth and proliferation and maintained cell viability to some extent under damaging conditions.

### 3.4. Small extracellular vesicles derived from hypoxic cultured UC-MSCs could repair mechanical damage to peripheral nerves

**FIGURE 4:**
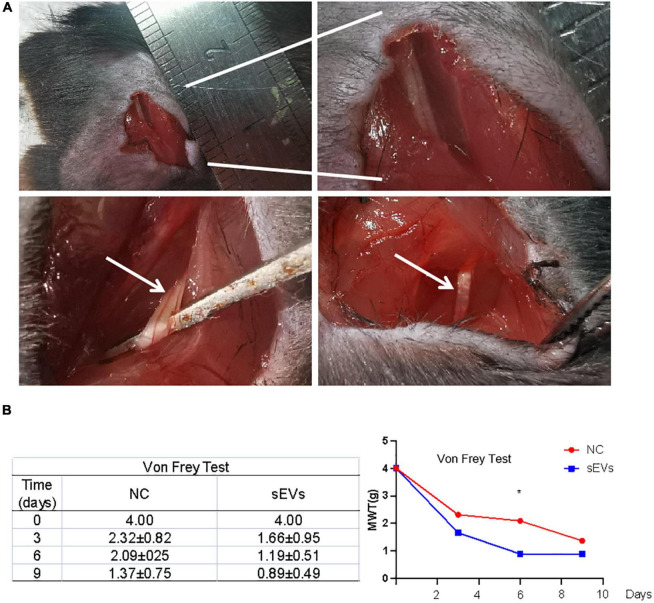
Monitoring of functional repair after sciatic nerve injury in mice. **(A)** A murine SNI model was established *via* open-field isolation of the mouse sciatic nerve and pressure-damaged sciatic nerve filaments. **(B)** Von Frey experiment: Paw withdrawal threshold was monitored to observe changes in pain threshold in control and sEV-treated mice. Statistical significance, *p* < 0.05. *Significant difference.

We designed a murine SNI model for functional validation in an *in vitro* experiment to verify the neuroprotective effect of hypoxic cultured UC-MSC-derived sEVs (Figure 4A). First, mice were anesthetized with 10% chloral hydrate, and the sciatic nerve was then isolated in an open field.

The SNI model mice were divided into two groups: Mice in the sEV-treated group were treated with 200 μL of sEVs resuspended in normal saline (1 mg/ml) for local infiltration and sutured; mice in the control group were treated with an equal volume of saline after injury and sutured.

After nine consecutive days of feeding, the affected limb of each mouse was subjected to the von Frey test (Figure 4B). The results showed that compared with those in the control group, the MWT values of the affected limb in the sEV-treated group exhibited fewer positive observations, indicating that the perceptual and feedback functions of the injured limb of the mice had been significantly repaired.

### 3.5. The effect of hypoxic cultured UC-MSC-derived sEVs in peripheral nerve repair is not dependent on the inhibition of inflammatory factor pathways

**FIGURE 5:**
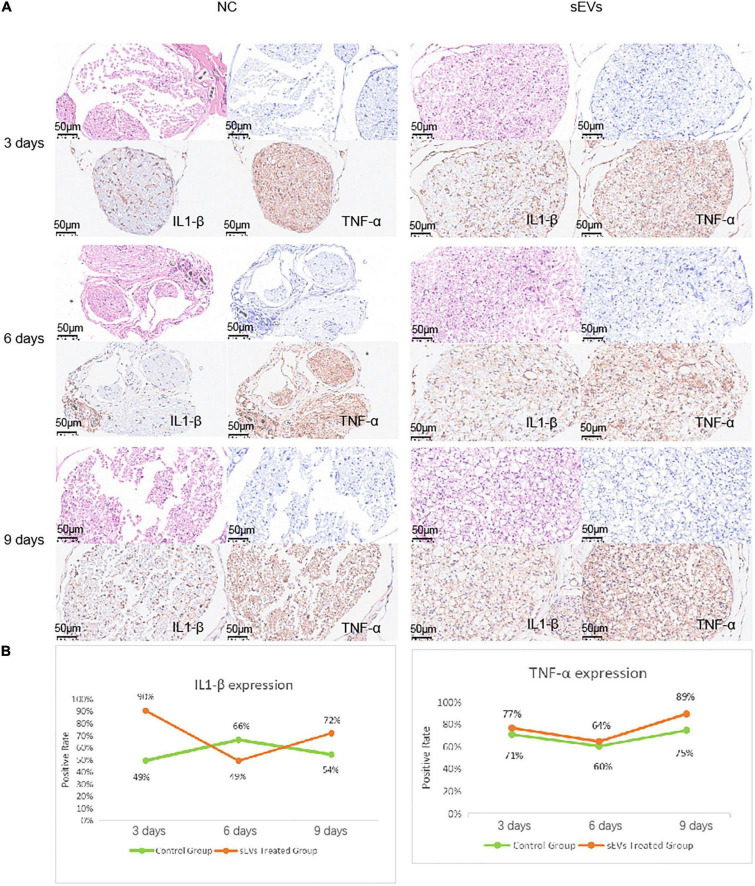
Sciatic nerve injury and pathological injury upon treatment. **(A)** Histological examination of sciatic nerve injury: Tissues from both the control and treated groups were sectioned at the 3-day interval to examine the extent of the injury. HE staining: deeply purple-stained nuclei and lightly stained pink cytoplasm. Toluidine blue staining: Darkly stained indigo nuclei. **(B)** Immunohistochemical analysis of IL1-β and TNF-α was carried out with antibodies (refer to note in the lower right corner), and the antibody dilution was set at 1:3,000 based on preliminary experimental conditions.

We performed functional tests and then collected sections of the injured tissue from sEV-treated and control mice for analysis. The number of cells in the myelin sheath was demonstrated by the density of HE and toluidine blue staining (Figure 5A). In the control sections, significantly more large cell-free areas were found, and this phenomenon decreased somewhat over time, but the gap in staining caused by cell death was clearly maintained at the injury site. Cell death was present in the intact myelin sheath of the sEV-treated mice, but the area was smaller than that in the control group. Observation of the sections on day 9 revealed that the number of nerve cells in the sEV-treated group was significantly higher than that in the control group. We stained for the inflammatory factors IL1-β and TNF-α and found that compared with the control group, the sEV-treated group consistently showed significantly increased positive staining (Figure 5B). The rate of tissue positivity in the control group gradually increased over time. Tissue TNF-α levels in the sEV-treated group were essentially the same on days 3 and 6 and then increased. Accordingly, we determined that hypoxic cultured UC-MSC-derived sEVs had a significant protective effect on cell division and the self-repair of injured peripheral nerve tissue in the mice, which was not dependent on the negative regulation of inflammatory factors. Instead, this effect may have been due to upregulated inflammatory factor expression, or other crosstalk mechanisms of regulation may have occurred.

## 4. Discussion

Peripheral nerve injury is a traumatic intractable condition with a high rate of incidence. Currently, the main focus is on the PNI and treatments used to repair and regenerate nerve tissue and reduction of neuroinflammation, as a result, creating a beneficial micro-environment for neurogenesis and axonal regeneration (Li F. et al., 2019; Yao et al., 2021). To date, great efforts have been made by researchers, but the effects of PNI treatment are still limited. MSC transplantation is promising in the treatment of PNI. Attempts at stem cell transplantation have shown some promise for the treatment of PNI (Erba et al., 2010; Wang et al., 2020; Zhang et al., 2021). MSCs are a useful tool for repairing both axonal and nerve damage (Sullivan et al., 2016). They are usually transplanted into the injured area as seed cells by local injection for therapy or along with tissue-engineered scaffolds. They are widely used in the treatment of nerve tissue regeneration (Cooney et al., 2016; Kizilay et al., 2017). The role of MSC transplantation has been demonstrated to promote axon regeneration and myelin formation and even to help recovery from denervated muscle atrophy. The effect is equivalent to direct transplantation of Schwann cells, which are difficult to regenerate and proliferate *in vitro* (Kim et al., 2018).

Small extracellular vesicles derived from MSCs are involved in many MSC biological processes and cell-to-cell communication, including the exchange of protein and nucleic acid information between donor and recipient cells (Sowa et al., 2016). In our sEVs uptake experiments, this characteristic was also intuitively and interestingly observed by the fluorescence tracer (Figure 2). In recent years, exosomes derived from bone marrow stromal cells (BMSCs) and adipose-derived stem cells (ADSCs) have been observed to promote PNI repair (Kourembanas, 2015; Liu C. Y. et al., 2020). However, the mechanisms for different stem cell-derived exosomes that contributed to PNI remain unclearly. Our study selected SCs as the key cells for PNI repair as the receptor cell model for exosome therapy. Results (Figures 2, 3) showed that UC-MSCs-sEVs could be taken into the cell by xenogeneic SCs to play a role. We observed that the uptake of exosomes by xenogeneic SCs directly promoted the proliferation of SCs, the function of sEVs replacing the role of MSCs transplantation treatment for nerve repair therapy as seed cells. In addition, the cytokine secretion and intercellular communication of SCs were changed, which may reveal the reason why UC-MSCs-sEVs can promote PNI repair.

The internal environment is closer to a hypoxic state. In hypoxic-related inflammatory diseases, exosomes promote immune responses mainly by mediating pro-inflammatory responses, and various mesenchymal stem cells and immune cell-derived exosomes show superior therapeutic potential in hypoxic-related diseases (Li D. et al., 2019; Kumar and Deep, 2020; Zhao et al., 2020; Xu et al., 2021). During the progress of UC-MSCs supernatant collection, the color of the supernatant in the hypoxic environment changed significantly, compared with the groups in normal culture, indicating that the PH of the supernatant was changed (results were not shown). The reason could be reported that the hypoxic precondition of MSCs can enhance their paracrine effects (Tirpe et al., 2019; Kang et al., 2021). Also, studies have shown that the expression of PI3K/Akt, nuclear transcription factor κB (NFκB), transforming growth factor β (TGF-β), and other signaling pathways change under hypoxia (Wozniak et al., 2017; Tirpe et al., 2019; Liu W. et al., 2020). In the effect of UC-MSCs-EVs with hypoxic pretreatment on Schwann cells, the data are shown in Figure 3, and the differentially expressed products were of great significance for this research. The differentially expressed products are of great significance for this project. Zeb2 is considered an inhibitor of negative regulators of Schwann cell development. Thus, the activation of Zeb2 by injury was an important feature of nerve cell repair (Houdhry and Harris, 2018). c-JUN was involved in SC reprograming and peripheral nerve injury response as a key transcription factor. The expression of the c-JUN gene increases rapidly after nerve injury and in peripheral nerve disease, and it has a critical role in axon regeneration (Quintes et al., 2016). MAPK pathway extracellular signal-regulated protein kinases (ERK 1/2) and c-JUN are activated after nerve injury and participate in myelin regeneration (Sheu et al., 2000; Nocera and Jacob, 2020). In our data, the expression of endogenous Zeb2, c-JUN, and ERK1/2 in SCs increased significantly after uptakes of hypoxic pretreated UC-MSCs-EVs by SCs. However, the protein expression level of SOX2 was not significantly increased in this experiment (the data were not shown). These results suggest that hypoxic preconditioning of UC-MSCs-EVs may promote nerve regeneration and myelin repair with endogenous activation in SCs.

To further investigate the effect of sEVs treatment on motor function improvement in animals, we examined the von Frey test and walking tracking test *in vivo* experiments. In the von Frey experiment: The paw withdrawal threshold was monitored to observe significant changes in pain threshold in control and sEV-treated mice. However, in the walking tracking test, four groups of mice (five mice in each group) were included in the experiment: Control group, injury group, injury assisted with saline treatment group, and injury assisted with sEVs treatment group, the difference between the treatment group and the control group was not significant (the data were not shown). The reason may suggest that the sEVs derived from mesenchymal stem cells can promote the repair of sciatic nerve injury to a certain extent, while the complete repair of the nerve and the recovery of motor function require sEVs derived from other different sources, such as Schwann cells, or the treatment with targeted drugs loaded with exosomes for nerve injury, such as miR-21 (Parkinson et al., 2008; Liu et al., 2022). Whether stem cells or exosomes, the mechanism of tissue repair is still unclear, which is the direction we will continue to study further.

For further analysis, the culture of UC-MSCs under hypoxic conditions slowed cell expansion after 72 h of culture, but the sEV content was significantly elevated. This suggests that hypoxia has a particularly important effect on sEV secretion, and the secretion profiles of sEVs produced by hypoxic stimulation may differ as a result. sEVs were taken up by wild-type SCs within 8 h, but the changes in nucleic acids and proteins at the cellular level were insignificant. This may be because the regulatory substances in sEVs do not directly regulate the corresponding functional factors but may be dependent on the presence of miRNAs, such as miRNA25-3p, which has recently gained attention, and the miRNA let-7b-5p, which has been shown to be involved in cellular repair processes in vascular injury, dry eye disease, and hepatitis, among others. The nucleic acids in sEVs are mainly miRNAs, which may also regulate crosstalk in the nerve injury repair process. *In vitro* experiments showed that SCs were protected to some extent after injury, confirming the presence of anti-injury and pro-repair functions in sEVs. In the *in vivo* experiments, the number of cells in the membrane sheaths was significantly higher in the sEV-treated group than in the control group, but the levels of IL1-β and TNF-α were significantly higher than those in the experimental group, which may be due to the apoptosis of sEV-assisted cells after anti-injury treatment. Therefore, the number of surviving cells after injury was high, and the functional base of cells that secrete inflammatory factors due to injury was large. sEVs prompted the secretion of inflammatory factors. This was dependent on stress feedback from the cells themselves to achieve a protective function. The protective function of sEVs did not depend on inflammatory factor secretion. As mentioned previously, high expression of inflammatory factors is a consequence rather than a cause of survival. sEVs’ regulatory functions depend on more complex crosstalk, such as crosstalk *via* small nucleic acids, for their realization. In conclusion, sEVs can serve as a promising therapeutic tool for PNI repair. The mechanism of hypoxic pretreated sEVs in PNI repair is worthy of further big data mining. The development of engineered exosomes and targeted drug delivery as a vector would be new possibilities for the treatment of PNI.

## Data availability statement

The raw data supporting the conclusions of this article will be made available by the authors, without undue reservation.

## Ethics statement

The animal study was reviewed and approved by the Animal Ethics Committee of the Peking University Health Science Center, China.

## Author contributions

All authors listed have made a substantial, direct, and intellectual contribution to the work, and approved it for publication.
